# The Influence of Collective Behavior on Pacing in Endurance Competitions

**DOI:** 10.3389/fphys.2015.00373

**Published:** 2015-12-09

**Authors:** Andrew Renfree, Everton Crivoi do Carmo, Louise Martin, Derek M. Peters

**Affiliations:** ^1^Institute of Sport and Exercise Science, University of WorcesterWorcester, UK; ^2^Department of Physical Education, Senac University CentreSao Paulo, Brazil; ^3^Faculty of Health and Sport Sciences, University of AgderKristiansand, Norway

**Keywords:** decision-making, endurance performance, complex systems, sport

## Abstract

A number of theoretical models have been proposed in recent years to explain pacing strategies observed in individual competitive endurance events. These have typically related to the internal regulatory processes that inform the making of decisions relating to muscular work rate. Despite a substantial body of research which has investigated the influence of collective group dynamics on individual behaviors in various animal species, this issue has not been comprehensively studied in individual athletic events. This is somewhat surprising given that athletes often directly compete in close proximity to one another, and that collective behavior has also been observed in other human environments including pedestrian interactions and financial market trading. Whilst the reasons for adopting collective behavior are not fully understood, collective behavior is thought to result from individual agents following simple local rules that result in seemingly complex large systems that act to confer some biological advantage to the collective as a whole. Although such collective behaviors may generally be beneficial, competitive endurance events are complicated by the fact that increasing levels of physiological disruption as activity progresses may compromise the ability of some individuals to continue to interact with other group members. This could result in early fatigue and relative underperformance due to suboptimal utilization of physiological resources by some athletes. Alternatively, engagement with a collective behavior may benefit all due to a reduction in the complexity of decisions to be made and a subsequent reduction in cognitive loading and mental fatigue. This paper seeks evidence for collective behavior in previously published analyses of pacing behavior and proposes mechanisms through which it could potentially be either beneficial, or detrimental to individual performance. It concludes with suggestions for future research to enhance understanding of this phenomenon.

## Introduction

“Pacing” is the term used to describe the distribution of muscular work rate throughout an exercise bout, and is a fundamental requirement of successful endurance performance (Foster et al., 1994). A great deal of published research in recent years has investigated the regulatory mechanisms that allow effective regulation of pacing to be achieved. Although there appears to be little consensus in the literature with regards to the precise processes involved, the momentary Rating of Perceived Exertion (Tucker, 2009), the Hazard Score (de Koning et al., 2011), and emotion (Baron et al., 2011; Renfree et al., 2012) have all been suggested to be contributing factors. More recently Smits et al. (2014) and Renfree et al. (2014) have identified the need for greater consideration of decision-making processes in explaining observed athletic behaviors. Again, whilst the precise processes remain unclear, several potential models have been proposed for further investigation. It is apparent, however that whilst considerable research effort has been invested in enhancing understanding of decision-making based on internal regulatory processes (Tucker, 2009; Marcora and Staiano, 2010; de Koning et al., 2011; Smits et al., 2014), less has been placed on the possible influence of external factors such as the relative presence, or indeed absence of other competitors. Collective behaviors have been described in a number of non-biological, animal and human environments, and can be explained by relatively simple laws governing interactions being followed by individual agents giving rise to complex large systems. The aim of this paper is to identify the possible mechanisms through which the presence of other competitors might influence collective group behavior and therefore individual pacing decisions, and to propose future research priorities.

## Collective behavior

A key feature of most individual competitive endurance events is that athletes race directly against other competitors, sometimes in individually marked lanes, and at other times within closer proximity to one another. This may mean that adopted behaviors are heavily influenced by those displayed by other nearby individuals, a phenomenon that has been studied extensively in other human and animal models. For example, so called “herd behavior” (Banerjee, 1992) has been found to occur in numerous situations. The model of herd behavior suggests that in complex decision-making environments, the “easiest” decision to make is simply to do exactly the same as those who happen to be in close proximity, or at least those of whom the individual is aware. Complex systems theory suggests that through individual agents following very simple local rules governing interactions, it is possible to generate large, seemingly complex patterns characteristic of biological systems (Wolfram, 1988). Through mathematical modeling, it has been demonstrated that individual agents following relatively simple rules can explain the collective motion (using terms such as swarms, schools, flocks, herds, and murmurations) of various animal species (King and Sumpter, 2012). A key feature of all these collective behaviors is that they emerge in the absence of any obvious centralized control, but rather because some localized information originating from neighbors flows through a system and results in the production of a collective pattern (Giardina, 2008). Although the precise reasons for the adoption of such behaviors are unknown, it is thought that they may aid in the avoidance of predation, or else be a mechanism through which useful information, such as location of food sources, may be conveyed between group members (King and Sumpter, 2012). Herd behavior has also been displayed by humans in various environments. For example, in financial markets individual market participants appear to mimic one another, leading to heavy tails in the distribution of stock price variations (Cont and Bouchaud, 2000), whereas self-organizing phenomena would appear to explain the “flow” behavior of pedestrians (Helbing et al., 2005), whereby the time gap between individuals is influenced by boundary conditions in corridors and at intersections. This tendency toward collective behavior and group formation appears to be based on a collective group memory, whereby previous history of group structure influences future collective behaviors, and individuals learn to change spatial positions within a group based on adoption of local “rules of thumb” (Couzin et al., 2002).

Interestingly, collective behavior appears to not only occur in biological systems. Experimental work by Giomi et al. (2013) demonstrated that brainless “bristle-bots” (constructed from toothbrush bristles and an on-board cell phone vibrator motor) transitioned to collective swarming and swirling behavior when confined to a limited area. This finding may suggest that the formation of collective behaviors is a spontaneous occurrence that translates into swarm intelligence. However, it must be acknowledged that while many analyses of collective behaviors have tended to treat individuals as simple interacting physical units (Giardina, 2008), there are potential limitations to this approach. Specifically, in biological systems individual behaviors may well-derive from complicated biological processes rather than simple physical laws. Indeed, and in relation to athletic activity, Smits et al. (2014) suggest that in order to fully explain decisions related to pacing in athletic events, it is necessary to understand how perception and action are coupled in determining behavior, therefore suggesting an ecological approach may be required.

## Collective behavior in sport

At this point it should be emphasized that competitive sporting events differ from most other human and animal environments in a key respect. Whilst the possible reasons for such behavior identified earlier, including avoidance of predation and the sharing of information relating to the location of food (King and Sumpter, 2012), may be expected to benefit the collective as a whole, in individual endurance events it would seem implausible that individuals would consciously adopt behaviors that would benefit other rival competitors. Competitive sporting events may therefore be considered rather artificial environments from a biological perspective, and the influence of engagement in collective behaviors warrants investigation. Given the complexity of the internal biological processes and the interactions between autonomous biological entities, identification of simple rules governing both individual and collective behavior in sport environments may be impossible. However, to our knowledge no study has attempted to identify relative weightings given to external and internal processes in determining decisions made relating to muscular work rate during individual competitive endurance events.

Although some research has suggested that sports teams should be considered “superorganisms” whose behavior results from collective processes (Duarte et al., 2012), less research is available relating to collective behavior in self-paced endurance activities. Undoubtedly any behavior displayed in such an environment would be complicated by the fact that performance capacity would be disrupted to a greater or lesser extent as an event progressed due to increasing physiological disruption. A financial trader or a pedestrian can “follow the herd” for long periods of time with few biological consequences, whereas a competitor in an endurance race may initially be able to do so before finding their ability to continue is compromised through metabolic disturbance. Indeed, in racing cyclists Trenchard et al. (2014) suggest a peloton exhibits collective behavior similar to that displayed by flocking birds or schooling fish. A number of general processes were proposed that explained the formation of large collectives and the separation of individuals or sub-groups from these during mass start velodrome races. These behaviors may reflect inherent evolved processes that maximize energy savings during collective activities. In a very recent paper, Trenchard (2015) goes on to suggest that cyclists display “protocoperative” behavior whereby they engage in cooperative activity. However, once the power outputs required for engaging in this activity become prohibitive due to continued physiological disruption, athletes can no longer cooperate, and eventually they become uncoupled from the peloton.

The issue of energy savings in cyclists described above may imply that collective behavior would be beneficial in endurance sports such as this where speeds are high. Indeed, a paper by Kyle (1979) suggests that 80–90% of the metabolic cost of cycling is accounted for by the overcoming of wind resistance, but that cycling in a group reduced power output required at typical racing speeds by 30%. Trenchard (2010) later suggested that the formation of the peloton, characteristic of cycle road races, is actually formed in order to maximize collective energy expenditure. During running, where speeds are considerably lower, Kyle (1979) found only 4–8% of total energetic expenditure was utilized in the overcoming of wind resistance, and this was reduced by just 2–4% when running in a group. If collective behavior is an evolved characteristic that informs decision-making in a group environments, then we propose that such behavior may indeed be detrimental to athletic performance in some sporting events (such as running races) in which high performance is not generally associated with any survival advantage (which would be the driver of evolved behaviors). In order to better understand the influence of collective behavior on pacing strategy then, it is necessary to seek evidence for this occurring in running events where it should be less advantageous from a physiological perspective.

## Evidence for collective behavior in competitive endurance events

There already exists some evidence for collective behaviors informing decisions relating to pacing during endurance events. In elite runners competing in both the World Cross Country Championships (Esteve-Lanao et al., 2014; Hanley, 2014) and the World Marathon Championship (Renfree and St Clair Gibson, 2013), a common observation was made in that all runners adopted similar absolute running speeds early in the races, but that runners who eventually finished behind the leading athletes progressively decelerated. This resulted in overall “positive” pacing strategies for the majority of athletes which are characterized by a second half completed at a slower speed than the first. Such strategies are typically considered suboptimal for events of this kind of duration (Abbiss and Laursen, 2008). In our analysis of the World Championship marathon race (Renfree and St Clair Gibson, 2013) we found the degree of underperformance depended on the athlete's absolute performance potential as determined by their personal best times over the distance. When all athletes were split into quartiles based on their eventual finishing position, it was not surprisingly found that mean personal best speeds of each quartile decreased from the leading athletes to those who finished toward the rear of the race. However, the degree of “underperformance” relative to personal best times also increased as athletes finished further behind the leaders. This would suggest that the adoption of collective behaviors (i.e., similar starting speeds) at the outset of the race had greater negative effects on the athletes with lower absolute performance capacities. Although no measures of physiological responses are available for this event, it can be speculated that physiological disruption would be greater in those athletes of lower performance capacity, and that therefore the degree of underperformance in the latter stages of the race would be greater. This disruption and underperformance may also be expected to result in higher ratings of perceived exertion and more negative affective responses. This may explain the findings by Mytton et al. (2015) who demonstrated that medal winning athletes in international running and swimming events displayed greater increases in speed in the final stages than non-medal winning athletes. This greater acceleration in pace would be possible as a result of the possession of a greater metabolic reserve capacity (Swart et al., 2009) in the superior athletes. Konings et al. (2015) also demonstrated very similar findings in 1500 m short track speed skaters, whereby “top” finishers were only faster than “bottom” finishers in the final 5 laps (out of 13.5) in elite level competitions. However, speed skating races are completed at higher speeds than running events of the same distance meaning that energy savings from collective behavior would be expected to be greater. Despite this, Konings et al. (2015) also found that tactical positioning during the latter stages of the race was a strong determinant of final finishing position. In this case then, it may be that the energetic costs of accelerating and overtaking leading athletes (and thereby skating further on the bends) may prohibit the gaining of positions when overall speeds are high, even though there may be benefits in avoiding leading earlier in the race. This example again emphasizes the importance of consideration of the behavior of other group members on explaining individual behaviors during competitive endurance events.

## Potential influence of collective behavior on mental fatigue

Although the above may suggest that collective behaviors may ultimately be detrimental to some individual athletes during events such as running races, it should be acknowledged that there are also potential benefits. Zouhal et al. (2015) found that drafting behind another runner improved 3000 m running performance without any reduction in energy expenditure or cardiovascular effort, leading the authors to propose that a pacemaker may act to improve performance through psychological mechanisms. It should however, be acknowledged that the data presented in this paper could also be interpreted in a different manner. An increased running speed at the same level of cardiovascular effort could also imply participants benefitted from an energy saving provided by drafting. Given that regulation of pace requires continual decision-making (Renfree et al., 2014; Smits et al., 2014), it may therefore be suggested that following another athlete may act by reducing the number of decisions to be made, and therefore decrease cognitive loading. Vohs et al. (2014) have established that the process of decision-making leads to a subsequent loss of self-control characterized by, amongst other things, reduced physical stamina and reduced persistence in the face of failure. Indeed, mental fatigue can be induced by prolonged periods of cognitive activity, and is associated with impaired exercise tolerance despite it not influencing cardiorespiratory or metabolic factors (Marcora et al., 2009). Some support for this suggestion that group membership may be beneficial in endurance events is provided by Hanley (2015) who analyzed pack running in the IAAF World half marathon championships. Those athletes who ran in packs throughout the race showed smaller decrements in speed than those who did not do so, or did so only for parts of the race. Those athletes who did run in packs throughout also demonstrated greater accelerations in pace in the final stages, suggesting either maintenance of a greater metabolic reserve capacity, or that they had developed lower levels of mental fatigue. Hanley (2015) went on to suggest that in order to optimize performance, athletes should identify likely rivals of similar performance capacity in advance of the race and then aim to run with them as part of their pre-race strategy. There is as yet, however, no evidence that this is actually a good strategy. If running as part of group is to be effective in maximizing endurance performance, its success or otherwise may therefore depend on the ability to accurately self-assess performance capacity and also that of other athletes. Any mismatch between individual physiological capacity and that of the group as a whole will lead to incomplete realization of performance capacity.

In contrast to endurance running races whereby athletes compete directly and in close proximity to one another, pool based swimming races are completed with athletes in their own individual lanes, meaning that collective behaviors are impossible. In swimming races pacing profiles are consistent between competitions, and elite athletes do not appear to vary their tactics or modify their pacing strategies between events (Skorski et al., 2014). Earlier work by Skorski et al. (2013) had also demonstrated that swimmers produced faster times in real than simulated competitions, and that these faster times were achieved through swimming faster in each intermediate stage rather than adoption of a different overall strategy. These observations may suggest that when athletes are isolated from their direct competitors as a result of swimming in their own lane, then the reduced opportunities to engage in collective behavior means there is less variation in pacing displayed by athletes of differing performance levels competing in the same event.

## Future perspectives

We have proposed that the human tendency toward collective behaviors may go some way to explaining pacing decisions displayed by competitive athletes in some athletic events. However, athletic events are rather “artificial” from a biological perspective, and therefore the effects of engagement in such behaviors are uncertain. Although this tendency may be advantageous in relatively high speed endurance sports whereby energy savings from drafting are significant (for example cycling), it may actually be detrimental in lower speed activities. Athletes with inferior physiological capacities will be unable to maintain work-rates set by superior athletes and consequently suffer both physiological and psychological perturbations. Indeed, although there is some evidence that athletes in running events of relatively long duration (cross country and marathon running) may select starting speeds based on those selected by other competitors, it may be hypothesized that the relative benefit of engagement in such collective behavior may be greater in shorter running events whereby potential energetic savings from drafting are increased. This could result in greater group density, or slower athletes maintaining contact with faster athletes for a greater fraction of total race distance. It may also be the case that collective behavior is less evident in sports where there is greater separation between athletes in space or else they are to some extent isolated from one another (for example through competing in their own lanes). Alternatively, it may be possible that engagement in collective behaviors could be beneficial to performance through reducing the requirement for continuous decision-making and a subsequent reduction in mental fatigue, even in activities where energetic savings through drafting are minimal.

Further research is required in order to better understand the relative influence of both internal (physiological) and external (environmental) variables on decision-making regarding work rate during self-paced competitive, individual endurance activity. This could eventually lead to the development of strategies that allow athletes to make better pacing decisions that may optimize physiological capacity. Additional work is also required to increase understanding of sport specific tactical issues that will allow individual athletes to make better pacing decisions that maximize their chances of optimizing performance potential.

## Author contributions

AR: Devising and drafting the study, and revising it critically for the intellectual content. EC: Devising and revising the study critically for the intellectual content. LM: Revising the study critically for intellectual content and final approval of the version to be published. DP: Revising the study critically for intellectual content and final approval of the version to be published.

### Conflict of interest statement

The authors declare that the research was conducted in the absence of any commercial or financial relationships that could be construed as a potential conflict of interest.
